# Socioeconomy as a prognostic factor for location of death in Swedish palliative cancer patients

**DOI:** 10.1186/s12904-021-00736-z

**Published:** 2021-03-14

**Authors:** Jonas Nilsson, Georg Holgersson, Gustav Ullenhag, Malin Holmgren, Bertil Axelsson, Tobias Carlsson, Michael Bergqvist, Stefan Bergström

**Affiliations:** 1 Center for Research & Development, Uppsala University/ County Council of Gävleborg, Gävle Hospital, Gävle, Sweden; 2 Department of Radiation Sciences Umeå University Hospital, Umeå, Sweden; 3 Department of Radiology, Gävle Hospital, Gävle, Sweden; 4 Department of Immunology Genetics and Pathology, Section of clinical and experimental oncology, Uppsala University Hospital, Uppsala, Sweden; 5 Department of Oncology, Uppsala University Hospital, 751 85 Uppsala, Sweden; 6 Department of Oncology, Gävle Hospital, Gävle, Sweden; 7 Unit of Clinical research center, Östersund, Sweden

**Keywords:** Location of death, Palliative care, Cancer patients, Register study, Socioeconomy

## Abstract

**Background:**

An important aspect of end-of-life care is the place of death. A majority of cancer patients prefer home death to hospital death. At the same time, the actual location of death is often against patient’s last-known wish. The aim of this study was to analyze whether socioeconomic factors influence if Swedish palliative cancer patients die at home or at a hospital. There is no previous study on location of death encompassing several years in Swedish cancer patients.

**Methods:**

Data was collected from the Swedish Register of Palliative Care for patients diagnosed with brain tumor, lung, colorectal, prostate or breast cancer recorded between 2011 and 2014. The data was linked to the Swedish Cancer Register, the Cause of Death Register and the Longitudinal Integration Database for health-insurance and labor-market studies. A total of 8990 patients were included.

**Results:**

We found that marital status was the factor that seemed to affect the place of death. Lack of a partner, compared to being married, was associated with a higher likelihood of dying at a hospital.

**Conclusion:**

Our findings are in line with similar earlier studies encompassing only 1 year and based on patients in other countries. Whether inequalities at least partly explain the differences remains to be investigated. Patients dying of cancer in Sweden, who do not have a life partner, may not have the option of dying at home due to lack of informal support. Perhaps the need of extensive community support services to enable home death have to improve, and further studies are warranted to answer this question.

## Background

An important aspect of end-of-life care is the place of death [1]. A majority of cancer patients across all socioeconomic groups prefer home death to hospital death [2, 3]. However, most deaths in developed countries occur in a hospital, and previous studies report that the actual location of death is often against patients last-known wish [4, 5]. As meeting patients’ choice of death place has been seen as a robust indicator of quality in end-of-life care, it is important to explore the issue and plan supportive care accordingly [6]. The likelihood of home death in comparison to hospital death seems to be greater among palliative cancer patients living in rural areas than those living in urban areas in Sweden. This geographical discrepancy can be explained, at least partly, by closer proximity to hospital emergency departments in larger cities than in the countryside. This explanation is in particular attributable to patients with acute conditions or refractory symptoms, which are common in specific cancer types such as lung cancer [7, 8]. Also, patients living in rural areas could be more likely to prefer home death since they would otherwise have to receive inpatient care far away from their families. Moreover, one previous Swedish study, focusing on 1 year only, has shown associations between socioeconomic factors (such as educational level and marital status) and place of death, indicating that socioeconomic inequalities influence end-of-life care [6].

The aim of the current study, which encompasses several years, is to analyze whether socioeconomic factors such as educational level, income and marital status influence if Swedish palliative patients die at home or at a hospital.

## Methods

### Data collection

As part of a doctoral project, data was obtained from the Swedish Register of Palliative Care (SRPC), which is a National quality register developed in 2005 and contains data that is collected retrospectively post mortem through a structured questionnaire that is filled out by responsible health care professionals, and includes thirty questions of interest in palliative care regarding the last week of life of the deceased. Data was collected for patients recorded from January 2011 through December 2014. Patients who were diagnosed and died due to cancer within the defined period were included. Record linkage was made between SRPC and the Swedish Cancer Register (SCR) by using the personal identity numbers assigned to all Swedish permanent citizens at birth [9]. Data was collected from patients with lung cancer (ICD, International Classification of disease, C34), high grade brain tumors (C71), prostate cancer (C61), breast cancer (C50) and colorectal cancer (C18, C19 & C20). Information on underlying cause of death was obtained from the Cause of Death Register (CDR) [10], coded according to ICD system. The coverage of reported events in CDR is comprehensive, estimated at more than 99% of all deaths [11]. Additional individual information regarding socioeconomic data was retrieved from the Longitudinal Integration Database for health-insurance and labor-market studies (LISA in the Swedish acronym) managed by Statistics Sweden, including data on income, education, employment, country of birth etc. [12]. Since there are only a few Hospices in Sweden, we have chosen not to involve them in the study.

### Definitions of socioeconomic data

Marital status was defined as married, unmarried, widow/widower or divorced at the year of death. Education level was stratified as low, middle or high. Low education level was defined as 9 years or less in compulsory school. Middle education level was defined as having attended secondary level school (Sw: gymnasium). High education level was defined as university studies. Income was defined as the disposable income (individualized by family) of the deceased at the year of death. Numbers are presented in thousands of Swedish crowns (SEK).

### Statistical analysis

Patients’ characteristics at baseline were presented with standard descriptive statistics. We used multiple logistic regressions to assess the relationship between socioeconomic factors and if patients died at home versus at a hospital. Our dependent binary variable equaled 1 if the individual had died at home and 0 if he or she died at a hospital. Our primary independent variables of interest were the patients’ marital status, education level, and disposable income (individualized by family). We also included two possible confounder variables: age and sex. Age and disposable income were treated as continuous variables and fitted by restricted cubic splines, as they tended to be non-linear. Adjusted odds ratios (aOR) and corresponding 95% confidence intervals (CI) were used as inference for all variables in the models. Confidence intervals that don’t overlap the null value indicate statistical significance. Data processing and statistical analysis were performed with the statistical software R.

## Results

**Fig. 1:**
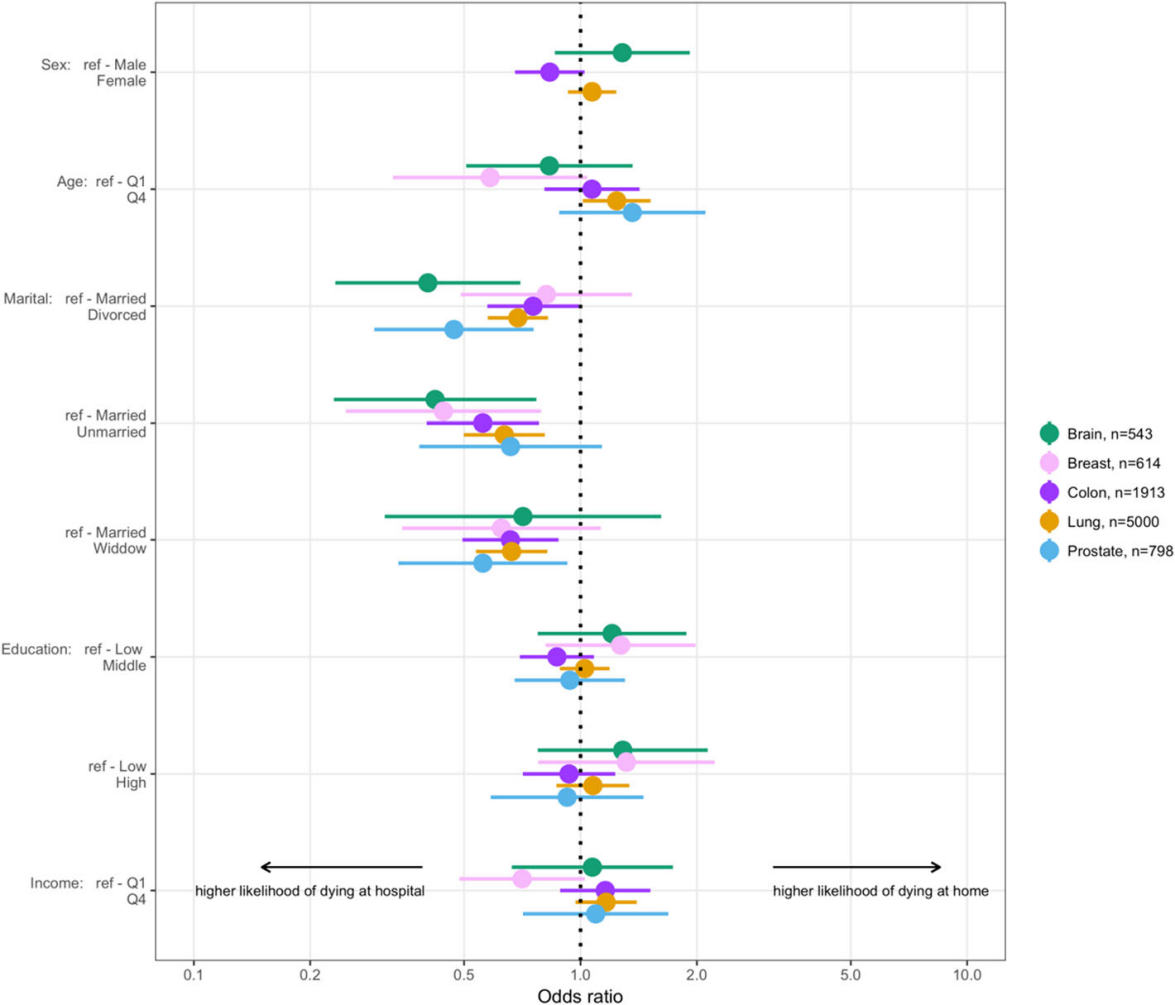
Odds ratios for dying at home vs dying at hospital for different tumor types

A total of 8990 patients were included in the study. Of these, 561 had a brain tumor, 621 had breast cancer, 1935 had colorectal cancer, 5062 had lung cancer and 811 had prostate cancer. For full demographic characteristics of the study population, see Table 1. For patients with lung cancer, the likelihood of dying at a hospital compared to dying at home was significantly higher for divorced (OR 0,69; 95% CI: 0.58–0.82), unmarried (OR 0,64; 95% CI: 0.50–0.81) and widowed (OR 0,66; 95% CI: 0.54–0.82) patients compared to married patients, holding all other predictors constant. For colorectal cancer, too, the likelihood of dying at a hospital compared to dying at home was significantly higher for divorced (OR 0,75; 95% CI: 0.57–0.99), unmarried (OR 0,56; 95% CI: 0.40–0.78) and widowed (OR 0,66; 95% CI: 0.49–0.88) patients compared to married patients. The same tendency was also seen for the other tumor types, although for brain tumors, the association with place of death was statistically significant only for divorced (OR 0,40; 95% CI: 0.23–0.70) and unmarried (OR 0,42; 95% CI: 0.23–0.77), for breast cancer only for unmarried (OR 0,44; 95% CI: 0.25–0.79), and for prostate cancer only for divorced (OR 0,47; 95% CI: 0.29–0.76) and widowed (OR 0,56; 95% CI: 0.34–0.92) when compared to married patients. There was a trend for higher likelihood of dying at home for patients with breast cancer and brain tumors with a high or middle education level compared to patients with a low education level. The differences were, not statistically significant, however, as the confidence intervals overlapped the null value. There was also a non-isolated trend for higher likelihood of dying at a hospital for patients with breast cancer with a high family income (fourth quartile) as compared to patients with a low family income (first quartile). For odds ratios for dying at home vs. dying at hospital for different tumor types, see Table 2. The findings are visualized in Fig. 1.

**Table 1:** Demographic characteristics of study population stratified by place of death

			Total, n (%)	Home, n (%)	Hospital, n (%)
Brain	Sex	Female	207 (100%)	92 (44.4%)	115 (55.6%)
		Male	354 (100%)	147 (41.5%)	207 (58.5%)
		NA	0	0	0
	Age	Median (Q1-Q4) min-max	65 (53–74) 0–89	65 (52–74) 5–89	65 (54–74) 0–89
		NA	0	0	0
	Marital	Divorced	78 (100%)	21 (26.9%)	57 (73.1%)
		Married	365 (100%)	176 (48.2%)	189 (51.8%)
		Unmarried	86 (100%)	29 (33.7%)	57 (66.3%)
		Widow	32 (100%)	13 (40.6%)	19 (59.4%)
		NA	0	0	0
	Education	Low	151 (100%)	56 (37.1%)	95 (62.9%)
		Middle	224 (100%)	100 (44.6%)	124 (55.4%)
		High	168 (100%)	73 (43.5%)	95 (56.5%)
		NA	18	10	8
	Income	Median (Q1-Q4) min-max	193.2 (126.76–312.32) -527.5-4372.9	192 (125.46–320.54) -527.5-1339.3	195.1 (130.34–307.34) -333.6-4372.9
		NA	12	7	5
Breast	Sex	Female	619 (100%)	174 (28.1%)	445 (71.9%)
		Male	2 (100%)	0 (0%)	2 (100%)
		NA	0	0	0
	Age	Median (Q1-Q4) min-max	69 (56–82) 27–100	66 (54–81) 33–94	69 (56–82.8) 27–100
		NA	0	0	0
	Marital	Divorced	108 (100%)	31 (28.7%)	77 (71.3%)
		Married	275 (100%)	93 (33.8%)	182 (66.2%)
		Unmarried	98 (100%)	19 (19.4%)	79 (80.6%)
		Widow	140 (100%)	31 (22.1%)	109 (77.9%)
		NA	0	0	0
	Education	Low	213 (100%)	53 (24.9%)	160 (75.1%)
		Middle	245 (100%)	74 (30.2%)	171 (69.8%)
		High	156 (100%)	44 (28.2%)	112 (71.8%)
		NA	7	3	4
	Income	Median (Q1-Q4) min-max	141.7 (103–214.8) -44.7-6627.6	135.3 (96.18–196.04) 0–590	143.4 (104.76–222.54) -44.7-6627.6
		NA	0	0	0
Colorectal	Sex	Female	927 (100%)	307 (33.1%)	620 (66.9%)
		Male	1008 (100%)	395 (39.2%)	613 (60.8%)
		NA	0	0	0
	Age	Median (Q1-Q4) min-max	74 (64–83) 15–99	73 (63–82) 22–97	74 (64–83) 15–99
		NA	0	0	0
	Marital	Divorced	316 (100%)	106 (33.5%)	210 (66.5%)
		Married	999 (100%)	415 (41.5%)	584 (58.5%)
		Unmarried	212 (100%)	62 (29.2%)	150 (70.8%)
		Widow	408 (100%)	119 (29.2%)	289 (70.8%)
		NA	0	0	0
	Education	Low	722 (100%)	263 (36.4%)	459 (63.6%)
		Middle	758 (100%)	265 (35%)	493 (65%)
		High	433 (100%)	165 (38.1%)	268 (61.9%)
		NA	22	9	13
	Income	Median (Q1-Q4) min-max	160.85 (118.16–249.22) -96-3319.7	166 (119.36–265.18) -96-1646.4	158.3 (117.7–241.74) 0.5–3319.7
		NA	1	0	1
Lung	Sex	Female	2391 (100%)	517 (21.6%)	1874 (78.4%)
		Male	2671 (100%)	595 (22.3%)	2076 (77.7%)
		NA	0	0	0
	Age	Median (Q1-Q4) min-max	71 (63.2–79) 14–97	72 (64–80) 14–95	71 (63–79) 23–97
		NA	0	0	0
	Marital	Divorced	1171 (100%)	216 (18.4%)	955 (81.6%)
		Married	2472 (100%)	627 (25.4%)	1845 (74.6%)
		Unmarried	589 (100%)	101 (17.1%)	488 (82.9%)
		Widow	829 (100%)	168 (20.3%)	661 (79.7%)
		NA	1	0	1
	Education	Low	2142 (100%)	465 (21.7%)	1677 (78.3%)
		Middle	2117 (100%)	462 (21.8%)	1655 (78.2%)
		High	742 (100%)	178 (24%)	564 (76%)
		NA	61	7	54
	Income	Median (Q1-Q4) min-max	157.3 (116.5–238.7) -369.8-4260	161.75 (118.38–255.1) -60.5-1901.1	156.4 (116.4–233.96) -369.8-4260
		NA	5	2	3
Prostate	Sex	Male	811 (100%)	295 (36.4%)	516 (63.6%)
		NA	0	0	0
	Age	Median (Q1-Q4) min-max	76 (68–84) 48–97	77 (68–84) 48–95	76 (68–84) 49–97
		NA	0	0	0
	Marital	Divorced	116 (100%)	28 (24.1%)	88 (75.9%)
		Married	517 (100%)	214 (41.4%)	303 (58.6%)
		Unmarried	79 (100%)	24 (30.4%)	55 (69.6%)
		Widow	99 (100%)	29 (29.3%)	70 (70.7%)
		NA	0	0	0
	Education	Low	370 (100%)	140 (37.8%)	230 (62.2%)
		Middle	287 (100%)	100 (34.8%)	187 (65.2%)
		High	141 (100%)	51 (36.2%)	90 (63.8%)
		NA	13	4	9
	Income	Median (Q1-Q4) min-max	173.6 (134.5–255.7) -0.6-6657.2	176.2 (136.04–252.72) -0.6-954.6	170.85 (134.4–257.7) 0–6657.2
		NA	0	0	0

**Table 2:** Odds Ratios for dying at home vs dying at hospital for different tumor types

		aOR (95% CI)
Brain, *n* = 543		
Sex	Male	Ref
	Female	1.28 (0.86–1.92)
Age	Q1	Ref
	Q4	0.83 (0.5–1.36)
Marital	Married	Ref
	Divorced	0.4 (0.23–0.7)^a^
	Unmarried	0.42 (0.23–0.76)^a^
	Widow	0.71 (0.31–1.62)
Education	Low	Ref
	Middle	1.24 (0.79–1.95)
	High	1.22 (0.75–1.99)
Income	Q1	Ref
	Q4	1.08 (0.67–1.75)
Breast, *n* = 614		
Age	Q1	Ref
	Q4	0.58 (0.33–1.04)
Marital	Married	Ref
	Divorced	0.81 (0.49–1.35)
	Unmarried	0.44 (0.25–0.79)^a^
	Widow	0.62 (0.34–1.12)
Education	Low	Ref
	Middle	1.28 (0.82–2.01)
	High	1.29 (0.76–2.17)
Income	Q1	Ref
	Q4	0.71 (0.49–1.03)
Colorectal, *n* = 1913		
Sex	Male	Ref
	Female	0.83 (0.68–1.02)
Age	Q1	Ref
	Q4	1.07 (0.81–1.42)
Marital	Married	Ref
	Divorced	0.75 (0.57–0.99)^a^
	Unmarried	0.56 (0.4–0.78)^a^
	Widow	0.66 (0.49–0.88)^a^
Education	Low	Ref
	Middle	0.87 (0.7–1.09)
	High	0.92 (0.71–1.21)
Income	Q1	Ref
	Q4	1.16 (0.89–1.52)
Lung, *n* = 5000		
Sex	Male	Ref
	Female	1.07 (0.93–1.24)
Age	Q1	Ref
	Q4	1.24 (1.01–1.52)
Marital	Married	Ref
	Divorced	0.69 (0.58–0.82)^a^
	Unmarried	0.63 (0.5–0.81)^a^
	Widow	0.66 (0.54–0.82)^a^
Education	Low	Ref
	Middle	1.02 (0.88–1.19)
	High	1.07 (0.87–1.32)
Income	Q1	Ref
	Q4	1.16 (0.97–1.4)
Prostate, *n* = 798		
Age	Q1	Ref
	Q4	1.36 (0.88–2.11)
Marital	Married	Ref
	Divorced	0.47 (0.29–0.76)^a^
	Unmarried	0.66 (0.38–1.14)
	Widow	0.56 (0.34–0.92)^a^
Education	Low	Ref
	Middle	0.94 (0.67–1.31)
	High	0.92 (0.6–1.42)
Income	Q1	Ref
	Q4	1.1 (0.71–1.69)

^a^statistically significant

An Odds ratio greater than 1 implies a higher likelihood of home death and one less than 1 implies a higher likelihood of in hospital deaths

## Discussion

In this register-based study we found that socioeconomic factors seem to have an impact on the place of death for all patient groups included, i.e. with brain tumors, breast, colorectal, lung and prostate cancer. Marital status was found to be the most important factor predicting place of death where the lack of a partner (i.e. being divorced, unmarried or widowed) was associated with a higher likelihood of dying at a hospital, compared to married patients. For income and education level the associations were not statistically significant, although patients with breast cancer and a high income tended to die at a hospital compared to those with low income.

The main strength of the present study is the large number of patients who have been identified through the SRPC, which covers a majority of patients who have died from a cancer diagnosis in Sweden. The SRPC has high validity but, nevertheless, a concern with all register-based studies is the quality of the register data. There are previous studies on cancer patients in other countries and one Swedish study which included patients from one single year. There is however no earlier study on Swedish patients encompassing several years. One remark about the data from the present study is the high representation of patients with lung cancer and the relatively low representation of patients with breast and prostate cancer, which are the two most common cancer types in Sweden. A possible explanation is that since only patients that were both diagnosed and died due to cancer during 2011 through 2014 were included, patients who survived more than 4 years after diagnosis, or were diagnosed prior to 2011 were not included. The relative 5-year survival of breast and prostate cancer is about 80–90%, whereas the relative 5-year survival rate for lung cancer is around 40%, and this difference could explain the relatively low percentage of breast and prostate cancer patients.

The impact of marital status on place of death in cancer patients has been studied previously with similar results as in the present study. In a study by Öhlén et al., associations between place of death and cancer types, and individual, socioeconomic and environmental characteristics were investigated for all cancer deaths in 2012 in Sweden [6]. Being married was associated with a higher likelihood of dying at home when compared to being unmarried, whereas education level did not seem to affect this likelihood. Cohen et al. have reported similar results on place of death in two studies with cross-sectional data from death certificates from various countries during 2002–2003 and 2008 [13, 14]. In both studies it was found that being married consistently increased the chances of dying at home in all countries. A higher education level was associated with a higher likelihood of dying at home in some of the countries, whereas in other countries the opposite was seen. In a study of factors affecting place of cancer death in London and New York City in the years 1995 through 1998 it was found that being in the lowest tercile of socioeconomic status as compared to the highest lowered the odds of death at home by 22% in London and 39% in New York City [15]. We did not find similar associations in our study. Perhaps the differences are at least partly explained by various socioeconomic conditions in different countries. Other studies have also found a significant association between having a partner and a higher likelihood of dying at home, whereas the relationship with income, occupation and education is less clear [16, 17].

In Sweden the bulk of health and medical costs is funded by regional and municipal taxes [18]. Thus the nominal cost for the individual patient is probably not an important factor in end of life care. In urban areas most of the home care is carried out by the regions, and in rural areas mainly as a cooperation between the regions/specialized palliative home care teams and nurses from the municipalities [19]. Nevertheless, home care is in principal generally available to Swedish cancer patients.

Having a partner might facilitate the home caregiving of the patient. Moreover, married people are probably more likely to have children, which further enhances the opportunities for informal support in end of life care. However, we don’t know the support-giving capabilities of family members, and we have not included if the patient’s need of external support such as home service and nursing visits was met. In addition, patients would probably not want to leave their loved ones and be cared for in a hospital. Further studies with interviews at the individual level should be conducted to answer these questions.

## Conclusion

We have conducted the first study encompassing several years on location of death in Swedish palliative cancer patients. Marital status was found to be the most important factor associated with place of death where the lack of a partner (i.e. being divorced, unmarried or widowed) was associated with a higher likelihood of dying at a hospital. Patients dying of cancer in Sweden, who do not have a life partner, may not have the option of dying at home due to lack of informal support. Perhaps community support services to enable home death have to improve, and further studies are warranted to answer this question.

## Data Availability

The dataset will not be published, since there are multiple variables that together could be used to identify individual patients. However data are available from the corresponding author on reasonable request.
